# Cognitive testing for dementia is adversely affected by administration in a foreign location

**DOI:** 10.1186/s13104-015-1021-3

**Published:** 2015-03-04

**Authors:** Cynthia Bechtel, Ruth Remington, Bruce Barton, Constance Barasauskas, Thomas B Shea

**Affiliations:** Framingham State University, Framingham, MA 01701 USA; Department of Quantitative Health Sciences, UMass Medical School, Worcester, MA 01655 UK; Department of Biological Sciences, UMass Lowell, Laboratory for Neuroscience, Lowell, MA 01854 USA

**Keywords:** Alzheimer’s disease, Cognitive testing, Orientation, Clinical trial

## Abstract

**Background:**

It is colloquially considered that cognitive tests can be adversely affected by administration in a foreign location. However, a definitive demonstration of this is lacking in the literature. To determine whether or not this is the case, we compared the results of cognitive testing in a familiar versus foreign environment by single test administrator of individuals diagnosed with Alzheimer’s disease randomized to placebo in a multi-site clinical study.

**Findings:**

Cognitive tests were administered to 6 long-term residents of an assisted living facility at their residence (the “Familiar” cohort). The identical tests were administered to a newly admitted resident and to 2 community-dwelling individuals who drove to the administrator’s office for the first time (the “Foreign” cohort). Secondary testing was administered 3 months later at the same respective locations. Caregivers of participants completed reports of mood, behavior and activities of daily living.

The Familiar cohort performed equally well at both visits. The Foreign cohort performed significantly worse than the Familiar cohort at baseline. They improved statistically, and matched Familiar cohort performance, by their second visit. Caregiver reports for both cohorts were unchanged between visits.

**Conclusions:**

These findings support the notion that a foreign location can adversely affect performance on cognitive tests, and therefore support cognitive testing in a familiar location.

## Findings

### Rationale

Cognitive impairment in Alzheimer’s disease encompasses disorientation, disorder confusion, and impairments in perception that can contribute to agitation [1-4]. In this regard, it is colloquially considered that cognitive screening and evaluation can be adversely affected by multiple distractors, among which is the administration of cognitive tests in a foreign location. However, we note that the literature is lacking in controlled analyses justifying whether or not this is the case. Towards this end, we herein present evidence demonstrating that conducting cognitive tests in an unfamiliar location can indeed adversely affect participant performance.

## Methods

These data were obtained by a single test administrator from one site of a multi-site clinical study for a nutritional intervention for Mild Cognitive Impairment and Alzheimer’s disease (ClinicalTrials.gov NCT01320527) [5]. The protocol was in compliance with the Helsinki Declaration and was approved by the New England IRB (Newton, MA). Signed consent forms were obtained from the participant and/or health-care proxy. Participants completed a test battery consisting of the Mini-mental State Exam (MMSE) [6,7], the Geriatric Depression Scale (GDS) [8,9], the Royal clock-drawing test (Clox 1 and Clox 2) [10], and the Dementia Rating Scale version II (DRS) [11-13]. Clock drawings were independently scored by two of us, with 100% inter-rater reliability. DRS scores as reported were adjusted for participant age and education (Age and Education Corrected Moan’s Scaled Score, or “AEMSS”; according to the DRS reference manual; www.psychassessments.com). Caregivers completed the Alzheimer’s disease Cooperative Society–Activities of Daily Living (ADCS-ADL) [14] and the Neuropsychiatric Inventory (NPI) [15] during the same test period.

These tests were administered to 6 long-term residents of an assisted living facility at their residential location. Tests were also administered to an additional resident in the same facility by this same test administrator; however, this testing session was held on the day after this participant was admitted to the facility. Two community-dwelling participants and their caregivers drove to the test administrator’s office for completion of the test battery. The 6 long-term residents are hereafter referred to as the “Familiar” cohort with reference to the familiarity of their surroundings during initial test administration; the newly-arrived resident and the 2 community-dwelling are referred to as the “Foreign” cohort with reference to the unfamiliarity of their surroundings during the initial test administration. Three months later, a second round of tests were administered in the same respective location as the first; the MMSE and GDS were intended for participant demographics and were completed only during the first testing session.

All participants had been randomized to placebo and therefore there was no direct intervention other than the potential placebo effect itself, which was in place for all of these participants. Their status as placebo recipients was determined only following code breaking according to trial protocol, which was subsequent to both visits as described above.

**Figure 1:**
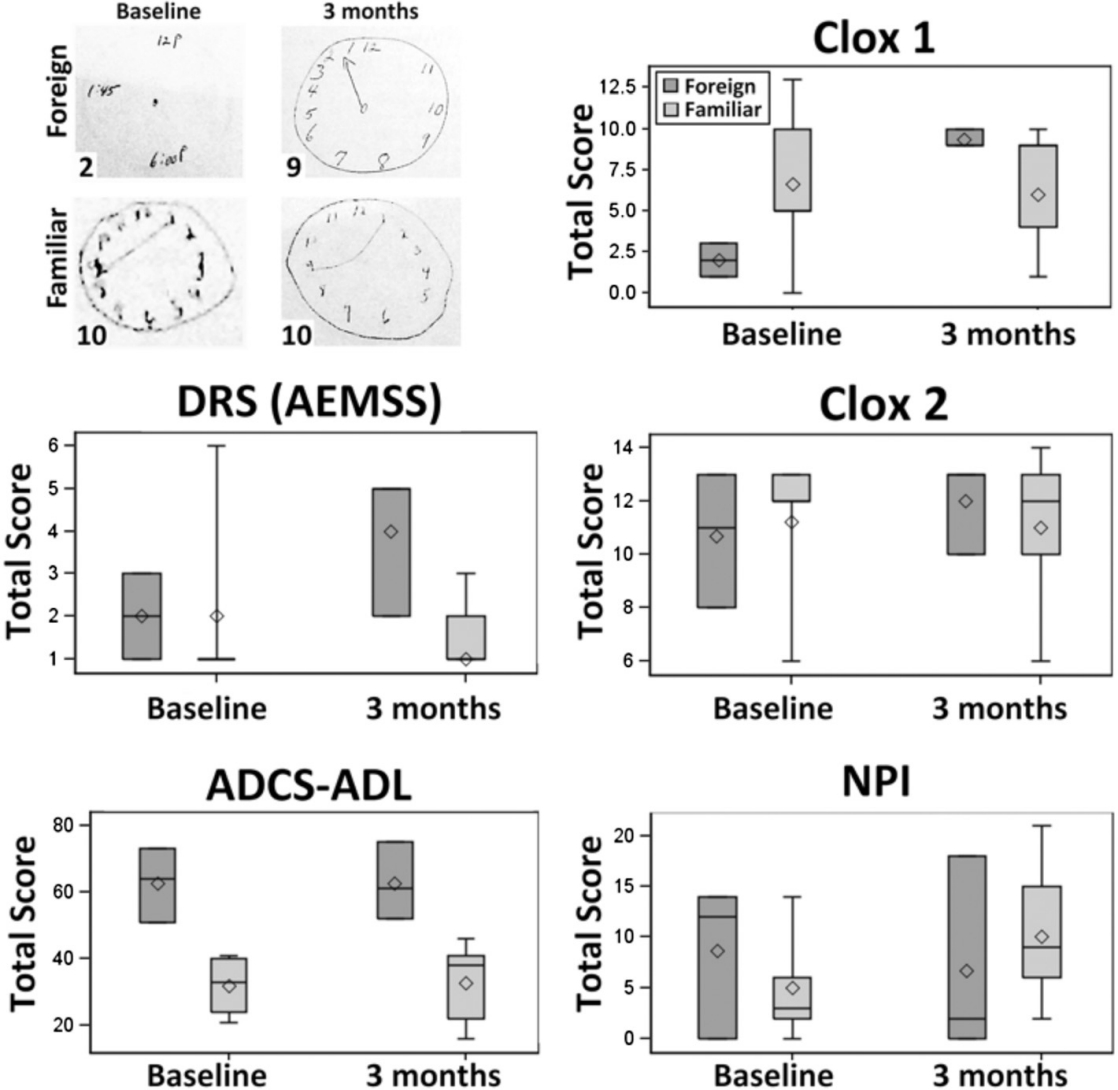
**Differential performance of foreign and familiar cohorts on the test battery.** Panels present representative Clox 1 images for Foreign and Familiar cohorts from the baseline and 3-month sessions as indicated. The respective scores out of the maximum possible 15 are indicated in the bottom left of each image. The accompanying graphs present the total scores both cohorts on the test battery; the boxes represent the 25^th^ and 75^th^ percentile (lower and upper ends, respectively), the line depicts the median, the diamond depicts the mean, and the whiskers represent the lowest and highest values that are not outliers. See text for further discussion.

Because of the small sample size, we used a non-parametric technique (i.e., Wilcoxon rank sum test) to compare the groups for baseline age, education, MMSE, and GDS as well as for the outcomes in Figure 1. For clarity, we presented the mean and standard deviation in Table 1 and standard box plots in Figure 1.

**Table 1:** **Demographics of participant cohorts**

	**Familiar**	**Foreign**	***p*** **value**
**Age**	87.5 ± 2.6	82.3 ± 5.8	0.22
**Education**	11.8 ± 0.4	12.3 ± 0.6	0.28
**MMSE**	18.7 ± 3.7	20.7 ± 4.7	0.76
**GDS**	3.7 ± 1.0	0.0 ± 0.0	0.03

Values represent the mean ± standard deviation. *P* values were determined between the Familiar and Foreign cohorts using a nonparametric Wilcoxon test.

No significant differences were noted in years of education and baseline MMSE scores between the Familiar and Foreign cohorts (Table 1). A trend towards significance was observed in average age; the average age of the Foreign cohort was 5 years younger than the Familiar cohort. However, the Foreign cohort displayed a significantly lower mean score on the GDS than did the Familiar cohort (Table 1).

## Results

At their initial session, the Foreign cohort demonstrated inferior performance (mean score of 2.0) as compared to that the Familiar cohort (mean score of 5.0) on Clox 1 (in which participants are asked to draw a clock that displays a specific time, with no clock to copy). Both cohorts performed equally well at the 3-month session (each with a mean score of 9.0; Figure 1). The change in performance of these cohorts between the baseline and 3-month test session differed statistically (p < 0.04; nonparametric Wilcoxon test). Performance of the Familiar cohort did not change between the baseline and 3 month session. The Foreign cohort did not display any corresponding impairment in performance on Clox 2 (which only requires copying an existing clock) nor any difference compared to the performance of the Familiar cohort on this test (Figure 1). The Foreign cohort did not display any corresponding impairment in performance on Clox 2 (which only requires copying an existing clock) nor any difference compared to the performance of the Familiar cohort on this test (Figure 1). The Foreign cohort displayed a doubling in scoring on the DRS between the initial and the 3-month session, while the Familiar cohort did not display any change in performance (Figure 1). Caregivers reported no change in the ACDS-ADL and the NPI (Figure 1).

Performance of the Familiar cohort on CLOX-1 was identical to that of the overall placebo group in the study from which these individuals were derived [5] both at baseline and after 3 months; the overall placebo group scored 7 ± 5 at baseline, and 7 ± 4 after 3 months; by contrast, the Foreign cohort scored over 2-fold lower than this overall placebo group at baseline, but scored identically to the overall placebo group after 3 months (Figure 1).

Examination of key portions of GDS and MMSE also supported the notion that an unfamiliar test location can adversely influence test performance. The Familiar and Foreign cohorts displayed identical scores (4.3 ± 0.3 and 4.3 ± 0.8, respectively, mean ± standard error of the mean) in response to questions on the MMSE regarding orientation, which have the potential to be adversely affected by testing in a novel location [16]. In addition, all 3 individuals of the Foreign cohort responded “no” to the GDS question, “Do you prefer to stay at home, rather than going out and doing new things?” By contrast, the majority (4/6) individuals in the Familiar cohort responded “yes” to this question. The observation that the Foreign cohort did not harbor a distinct lack of orientation or a unique adversity to traveling to a novel location further indicates that the novel location of baseline testing exerted an acute adverse effect on their cognitive performance.

## Discussion and conclusions

In the absence of any intervention, the significantly-improved performance of the Foreign cohort at their second session, coupled with the lack of change in the Familiar cohort, suggests that the performance of the Foreign cohort was adversely affected by the initial unfamiliarity of the test location. In support of this notion, the Foreign cohort did not display any corresponding impairment in performance on Clox 2 (which only requires copying an existing clock) nor any difference compared to the performance of the Familiar cohort on this test (Figure 1).

Further support was derived from caregivers, who reported no change in the ACDS-ADL and NPI (each of which reflect day-to-day performance at the individuals’ residence and are therefore independent of test location). Moreover, caregivers indicated that the Foreign cohort displayed an average statistically superior performance in the ACDS-ADL versus the Familiar cohort (p < 0.04 for both test sessions). This is perhaps to be expected since two individuals of the Foreign cohort were still community-dwelling and the third had just been admitted to the assisted living facility, while all members of the Familiar cohort were established residents of the assisted living facility; Nevertheless, superior performance of the Foreign versus Familiar group on the ADCS-ADL is consistent with conclusion that the lower cognitive performance of the Foreign cohort scores at the baseline visit was a result of conducting these tests in an unfamiliar location.

The limited findings presented herein with this small cohort of participants provide evidence supporting the need for a consist location for administration of cognitive testing, and suggests that failure to do so may increase the likelihood of aberrant scores, which could artifactually lower the mean performance of their cohort. The use of a small sample size imposition a major limitation on interpretation of our findings. However, we note that these findings were serendipitous, and, furthermore, we are unable to find any comparable report in the literature. We therefore consider the presentation of these findings important to carry the notion of avoiding a foreign location beyond colloquial considerations. The use of the same administrator in the second round of testing may also have contributed to increased familiarity and therefore improvement in scores. Another limitation, somewhat related to our small test population, is the difference (although not statistically significant) in age ranges of our two cohorts (Table 1).

Since even simple disruptions in routine can confound individuals with dementia, including even carrying out cognitive testing at different times during the day [17], it is not surprising that testing in a novel location may adversely influence test results. These findings address an inherent difficulty in clinical studies of early dementia, where a substantially greater number of participants are likely to be community-dwelling, while those in later stages are more likely to reside in assisted living facilities or nursing homes. Cognitive testing of residents of nursing homes/assisted living facilities is more likely to be perceived as relatively routine, since residents commonly encounter facility staff within their residence. By contrast, cognitive testing of community-dwelling individuals poses the inherent difficulty of requiring either a home visitation, which may seem invasive to the participant as well as the caregiver, and may prompt or necessitate traveling to a novel location for test administration. Notably, since our Foreign cohort displayed significantly improved performance at the second testing session, which represented only the second time that the 2 community-dwelling participants had travelled to this location, we note that one or two visits to the planned testing location, with either no testing or perhaps a simple introductory session, may be useful to reduce the potential adverse influence of a foreign location on cognitive evaluation. Similarly, our test administrator was unfortunately not made aware that the resident of the assisted living facility in our Foreign cohort had been admitted the previous day; the significantly improved performance of this individual on the second testing session clearly indicates that newly-admitted individuals should be allowed an orientation period prior to cognitive evaluation to gain familiarity with the novel residence.
